# Stentless aortic valve replacement in the young patient: long-term results

**DOI:** 10.1186/1749-8090-8-68

**Published:** 2013-04-08

**Authors:** Torsten Christ, Herko Grubitzsch, Benjamin Claus, Wolfgang Konertz

**Affiliations:** 1 Department of Cardiovascular Surgery, Charité-Universitätsmedizin Berlin, Charitéplatz 1, 10117 Berlin, Germany

**Keywords:** Aortic valve, Aortic valve disease, Aortic valve replacement, Stentless aortic valve replacement, Valve replacement

## Abstract

**Background:**

Stentless aortic valve replacement (SAVR) became a common surgical procedure to treat aortic valve disease, as it offers larger orifice area and improved hemodynamics. The aim of our single-centre retrospective study was to assess long term results of first generation stentless aortic valves in young patients, where mechanical prostheses are considered first line therapy.

**Methods:**

From 1993 to 2001, 188 (149 male and 39 female) patients (≤60 years) underwent SAVR. Indications were in 63.3% stenosis or mixed lesions and in 36.7% isolated regurgitation. Mean age of patients at surgery was 53.1 ± 7.1 years. Associated procedures were performed in 60 patients (31.9%). Follow-up data were acquired through telephone interviews. Follow-up was 90.4% complete at a mean of 8.8 ± 4.7 years. Total follow-up was 1657.6 patient-years with a maximum of 17 years.

**Results:**

Overall hospital mortality was 3.2% (2.5% for isolated SAVR). Overall actuarial survival-rate at 10/15 years and freedom from reoperation at 10/14 years were 73.0% ± 3.5%/ 55.8% ± 5.4% and 81.0% ± 3.4%/ 58.0% ± 7.5%, respectively. For isolated SAVR, actuarial survival at 10/15 years and freedom from reoperation at 10/14 years were 70.1% ± 4.4%/ 64.1% ± 4.8% and 83.1% ± 4.0%/ 52.9% ± 9.0%, respectively. Reoperation was performed in 42 patients (22.3%) due to structural valve deterioration and endocarditis. Age (≤50 years) and associated procedures did not significantly lower survival and freedom from reoperation, however, small prosthesis sizes (≤25 mm) did.

**Conclusion:**

In patients aged 60 and younger, SAVR provides reliable long-term results especially for larger aortic valves.

## Background

Stentless aortic valve replacement has become a common surgical treatment of aortic valve disease. These prostheses are designed to allow physiological flow patterns by avoiding an obstructive stent [1]. Various studies revealed controversial results comparing stented and stentless prostheses. However, improved hemodynamics and a larger orifice area could be shown in a recent meta-analysis [2]. In a prospective randomized trial, SAVR showed a midterm survival advantage compared to stented aortic valve replacement (AVR) [3].

Implantation of bioprosthetic aortic valves is still controversial in younger patients. The American Heart Association and the American College of Cardiology recommend bioprosthetic aortic valves in patients above the age of 60 years, after lowering the age recommendation down from 65 years in 2010 [4]. Due to their advanced hemodynamics, SAVR might have a better longevity and therefore may be used also in younger patients. The aim of our single-centre retrospective study was to assess the first very long-term results of SAVRs in patients ≤60 years.

## Patients and methods

From 1993 to 2001, 839 patients underwent SAVR. After excluding patients with endocarditis, dissection of the ascending aorta and over the age of 60 years, 188 patients for this study were identified. Either the patient’s explicit desire for bioprosthetic AVR or a contraindication to oral anticoagulation caused a decision for SAVR.

Mean age of patients at surgery was 53.1 ± 7.1 years. Baseline preoperative characteristics are presented in Table 1. Different types and sizes of prostheses used are shown in Table 2. Isolated SAVR was performed in 120 patients. Associated procedures were performed in 68 patients (36.2%) and are presented together with operative data in Table 2.

**Table 1:** Baseline characteristics

**Characteristic**	**Number**	**Percent**
Number of patients	188	
Mean age	53.1 years	
Mean standard error	7.1 years	
Range	24 – 60	
Sex		
Male	149	79.3%
Female	39	20.7%
Age		
> 50 years	145	77.1%
≤ 50 years	43	22.8%
Left ventricular function		
Normal	109	58%
Moderately impaired	58	30.8%
Profoundly impaired	21	11.2%
Aortic valve lesion		
Stenosis	62	33.0%
Insufficiency	69	36.7%
Mixed lesion	57	30.3%
Timing of operation		
Elective	185	98.4%
Urgent/ emergency	3	1.6%
Ascending aortic aneurysm	21	11.2%
Coronary artery disease	18	9.6%

**Table 2:** Operative data

**Procedure**	**Number**	**Percent**
Isolated SAVR	128	68.1%
SAVR + other	60	31.9%
AscAo	21	11.2%
MVR	16	8.5%
CABG	13	6.9%
CABG + other	5	2.6%
Myectomie	3	1.6%
Closure of VSD	1	0.5%
Passive Cardiomyoplasty*	1	0.5%
Surgical approach		
Median sternotomy	182	96.8%
Upper ministernotomy	6	3.2%
Implanted valves		
Edwards Prima-Plus®	120	63.8%
SJM Toronto-SPV®	50	26.6%
Medtronik FreeStyle®	10	5.3%
Vascutec Elan®	7	3.7%
Shelhigh-stentless®	1	0.5%
Implanted valve sizes		
23 mm	3	1.6%
25 mm	35	18.6%
27 mm	79	42.0%
29 mm	71	37.8%

AscAo = Replacement of ascending aorta; VSD = ventrical septal defect; CABG = coronary artery bypass grafting; MVR = mitral valve repair/ replacement; * = ACORN® Cardiac Support Device.

Follow-up data were acquired through telephone interviews. Follow-up was 90.4% complete at a mean of 8.8 ± 4.7 years. 21 patients were lost to follow-up. Total follow-up was 1657.5 patient-years with a maximum of 17.0 years. Data collection and statistical analyses were done according to the current guidelines for reporting mortality and morbidity after cardiac valve interventions [5]. The study was approved by the local Ethics Committee (Ethikkommission der medizinischen Fakultät der Charité - Universitätsmedizin Berlin).

### Statistical analysis

All data were analyzed with PASW Statistics version 18.0.0 (SPSS Inc., Chicago, Illinois). Descriptive statistics are reported as the mean ± standard deviation for continuous variables and as frequencies and percentages for categorical variables, unless otherwise noted. Survival and time-to-event analyses were performed using Kaplan-Meier actuarial methods. Age-stratified curve comparisons were performed using the log-rank test. All p values were two-sided. In addition, proportional-hazard models were used to investigate the following variables as risk factors for survival and freedom from reoperation: gender, age, left ventricular ejection fraction, aortic valve lesion, associated procedures, SAVR-diameter. Left ventricular ejection fraction was assessed by semi-quantitative definitions (normal, moderately impaired, profoundly impaired) due to bias because of the long period of follow-up (involving various physicians and different cardiovascular ultrasound systems). Statistical significance was set at a p value of less than 0.05. Preoperative only very few patients showed impaired renal function, diabetes, and essential hypertension (probably due to the low age of the patients) and consequently these characteristics were not entered as covariates in the analysis.

Age- and gender-matched survival estimates from the general German population were obtained from the Human Lifetable Database [6]. Age- and gender-specific conditional probabilities of surviving a 1-year age interval were used to create an age- and gender-matched patient sample. The survival line depicted in the figure comparing the general population with our study cohort represents these averaged conditional probabilities of survival.

## Results

### Hospital mortality

Overall hospital mortality was 3.2% (n = 6). Causes for hospital mortality were left ventricular failure (n = 3), right ventricular failure (n = 1), electromechanical dissociation (n = 1) and multi organ failure (n = 1). For isolated SAVR hospital mortality was 2.5% (n = 3).

### Patient survival

Overall actuarial survival at 10 and 15 years were 73.0% ± 3.5% and 55.8% ± 5.4%, respectively. For isolated SAVR actuarial survival at 10 and 15 years were 70.1% ± 4.4% and 64.1% ± 4.8%. During follow-up 61 patients died. Linearized mortality rate is 3.6%/ year for overall SAVR and 3.4%/year for isolated SAVR. Clinical follow-up of the patients with the originally implanted valve still in place showed only 1 patient in NYHA stadium III and no patient in NYHA stadium IV.

Univariate statistical analyses revealed no significant survival difference between isolated SAVR and combined procedures. Neither patients aged ≥50 years and patients aged <50 years, nor patients with isolated insufficiency as indication for operation and those with a stenotic valve lesion showed a significant difference in comparison. Additionally also the different models of SAVR did not influence survival.

**Figure 1:**
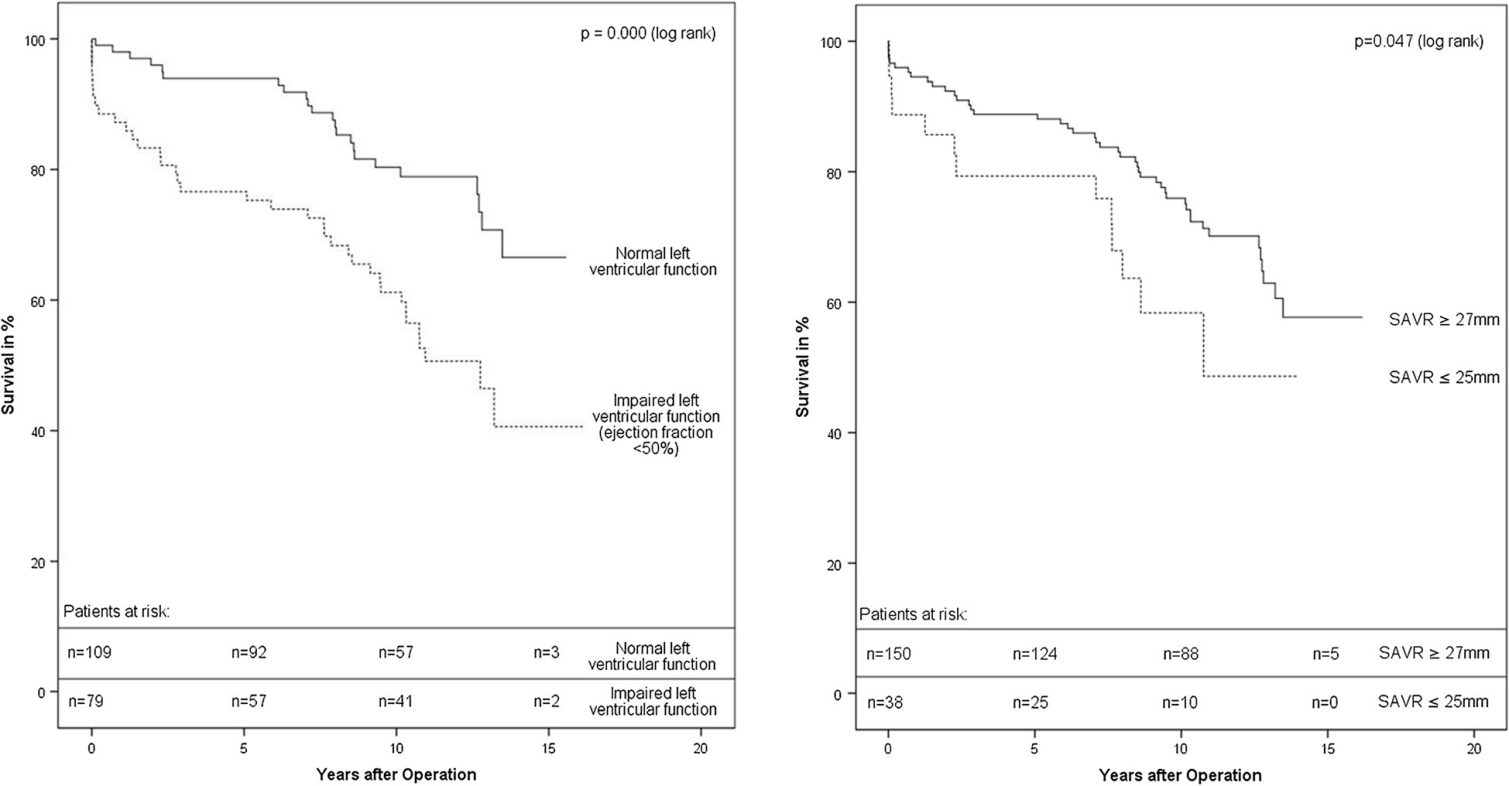
Survival after SAVR (Impact of left ventricular function and size of SAVR).

Various other factors had a significant impact on survival in univariate statistical analysis. Impaired left ventricular function (left ventricular ejection fraction ≤ 50%) significantly lowered survival. Patients with impaired left ventricular function showed an actuarial survival-rate at 10 and 15 years of 61.2% ± 5.7%/ 40.6% ± 8.1% (Figure 1), respectively. Besides this, patients with larger prosthesis sizes (≥27 mm) compared with small prosthesis sizes (≤25 mm) showed a significant survival benefit (Figure 1).

The multivariate proportional-hazard model supports left ventricular function and prosthesis size to be independent risk factors. Additional independent risk factors were age and isolated insufficiency of the aortic valve. (Table 3).

**Table 3:** Risk factors for mortality and reoperation

		**Survival**		**Freedom**	**from**	**Reoperation**
**Characteristic**	**HR**	**C I**	**Significance**	**HR**	**C I**	**Significance**
Age (≤50)	2.55	1.13-5.75	0.02	0.98	0.45-2.11	0.96
Gender	1.21	0.57-2.64	0.61	1.95	0.84-4.53	0.12
Aortic valve lesion (stenosis vs. insufficiency)	0.55	0.31-0.95	0.03	0.74	0.35-1.57	0.44
Left ventricular function (normal vs. impaired)	0.39	0.22-0.68	0.01	1.08	0.55-2.15	0.05
Associated procedures	0.94	0.52-1.70	0.83	0.54	0.23-1.25	0.15
SAVR Diameter (≤25 mm vs. >25 mm)	2.15	1.13-4.09	0.02	3.53	1.57-7.92	0.02

CI = Confidence Interval, HR = Hazard ratio.

### Freedom from reoperation

Reoperation was performed in 42 patients (22.3%). Causes were structural valve deterioration (83.3%) and endocarditis (16.7%). Structural valve deterioration caused in most cases (85.7%) insufficiency and in 14.3% stenosis.

Overall freedom from reoperation at 10 and 14 years was 81.0% ± 3.4% and 58.0% ± 7.5%, respectively. For isolated SAVR freedom from reoperation at 10 and 14 years was 83.1% ± 4.0% and 52.9% ± 9.0%, respectively. Linearized reoperation rate is 2.8%/year.

No significant statistical difference was found between isolated SAVR and combined procedures. Additionally also the different models of SAVR did not significantly influence freedom from reoperation.

**Figure 2:**
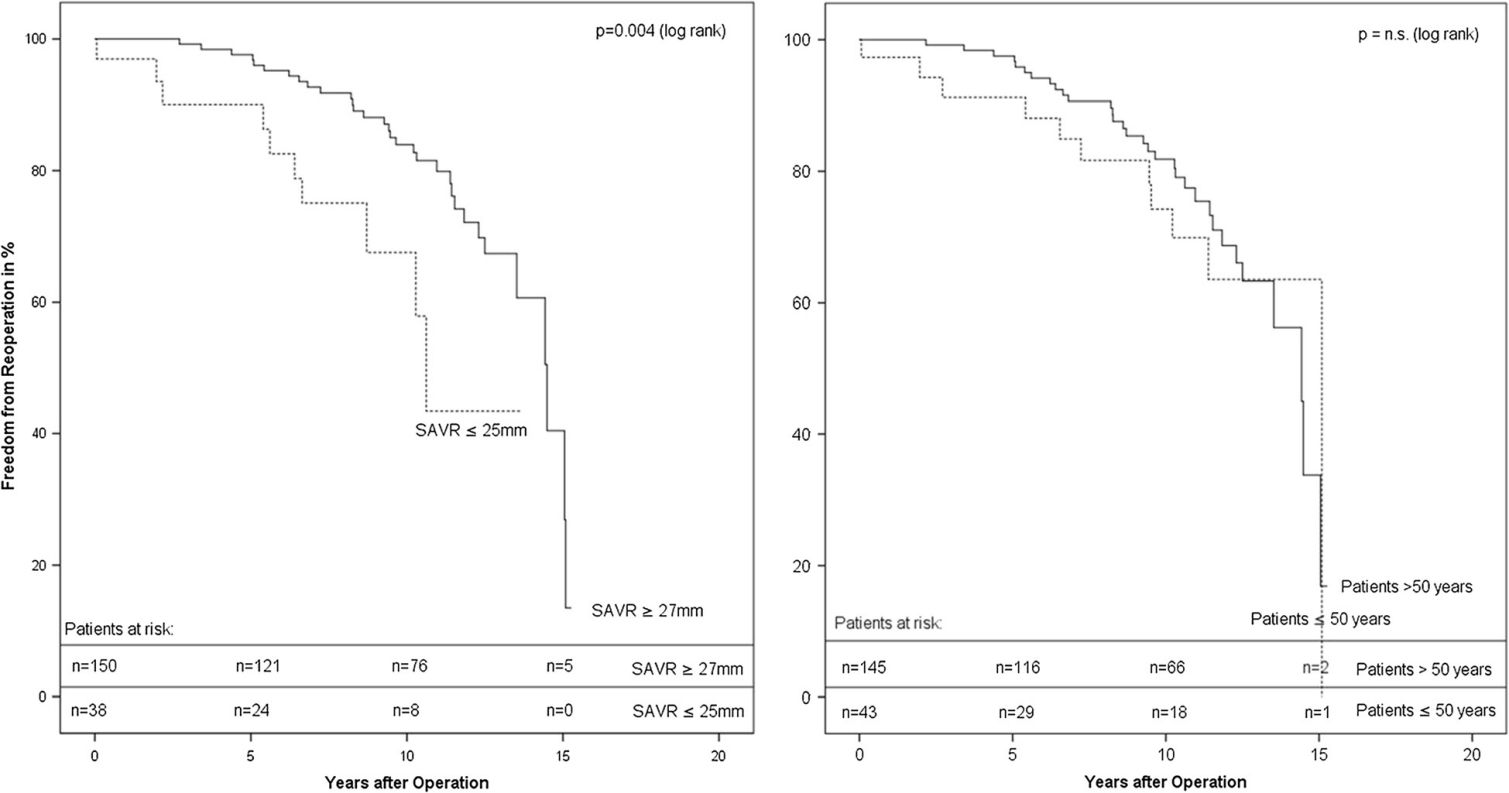
Freedom from Reoperation (Impact of prosthesis-diameter and patients age).

Noteworthy, in univariate statistical analysis only small prosthesis size (≤25 mm) significantly lowered freedom from reoperation, whereas age (≤50 years), aortic valve lesion and combined procedures had no significant influence (Figure 2).

The multivariate proportional-hazard model also revealed only prosthesis size as an independent risk factor (Table 3).

## Discussion

Nonrandomized and randomized clinical trials show evidence that SAVR provides better hemodynamic performance than stented AVR [1,2,7]. This advantage could reduce operative mortality, particularly in those with impaired left ventricular function. Late mortality could be decreased by better left ventricular wall mass regression and performance [2,8].

To compare this study to others is challenging. Most other trials deal with cohorts of patients with far older age. Besides this, our cohort showed a high percentage (42%) of patients with impaired left ventricular function (Table 1) with the inherent risk of premature death. Isolated insufficiency as cause of AVR is well presented in our cohort. Inferior early and long term survival has been shown for those patients, especially in younger age [9,10].

In 2010, McClure et al. published a late follow up after implantation of Carpentier-Edwards pericardial AVR. Overall actuarial survival at 10 and 15 years for patients less than 65 years of age was 71.5% and 43.7% [11]. The study population underwent more combined procedures and had fewer patients with impaired left ventricular function and insufficiency as cause of operation. Assuming the cohort had the same mean age as ours (no mean age was published) we see a more than 10% better survival rate after SAVR in our cohort.

Valfré et al. published a 25 year follow up of the Hancock II porcine valve. Their patient cohort had less comorbidities and received less combined procedures. Patients under 60 years showed a 10/15 year survival of 69.4/60.0% [12]. Because no mean age of study population was given, one can only assume comparable long term results.

Ruel et al. described a survival at 10 and 15 years of 82% and 70% in patients with a mean age of 47.6 years [13]. They had fewer patients with impaired left ventricular function and less combined procedures. With a 5.5 years older study cohort (in general population this equals a decline in 15 year survival of around 8%) our data suggest comparable long term results.

The only randomized prospective trial dealing with patients of almost the same mean age as ours, published by Oxenham et al., compares Bjork-Shiley mechanical valves with porcine AVR. A 10 year survival of 65.7% for isolated AVR in this cohort is well below our survival rate [14]. One has, however, to consider that those operations were performed in the 1970s.

Several randomized trials showed no significant survival difference between mechanical AVR and bioprosthetic AVR [14,15]. However, Ruel et al. showed a (nonsignificant) tendency of better survival towards bioprostheses of around 13% at a 20 year follow-up and of around 10% at 25 year follow-up [13]. Above that, they also found a significant difference in freedom from death after 20 years, attributable to ischemic or hemorrhagic stroke, with 97.9 ± 1.2% in tissue AVR patients and 83.9 ± 4.9% in mechanical AVR patients [13]. Despite lifelong need for anticoagulation treatment and elevated risk of bleeding [14], the risk of thrombembolic complications ranges between 0.7%-3.0%, depending on valve type and other risk factors [16].

**Figure 3:**
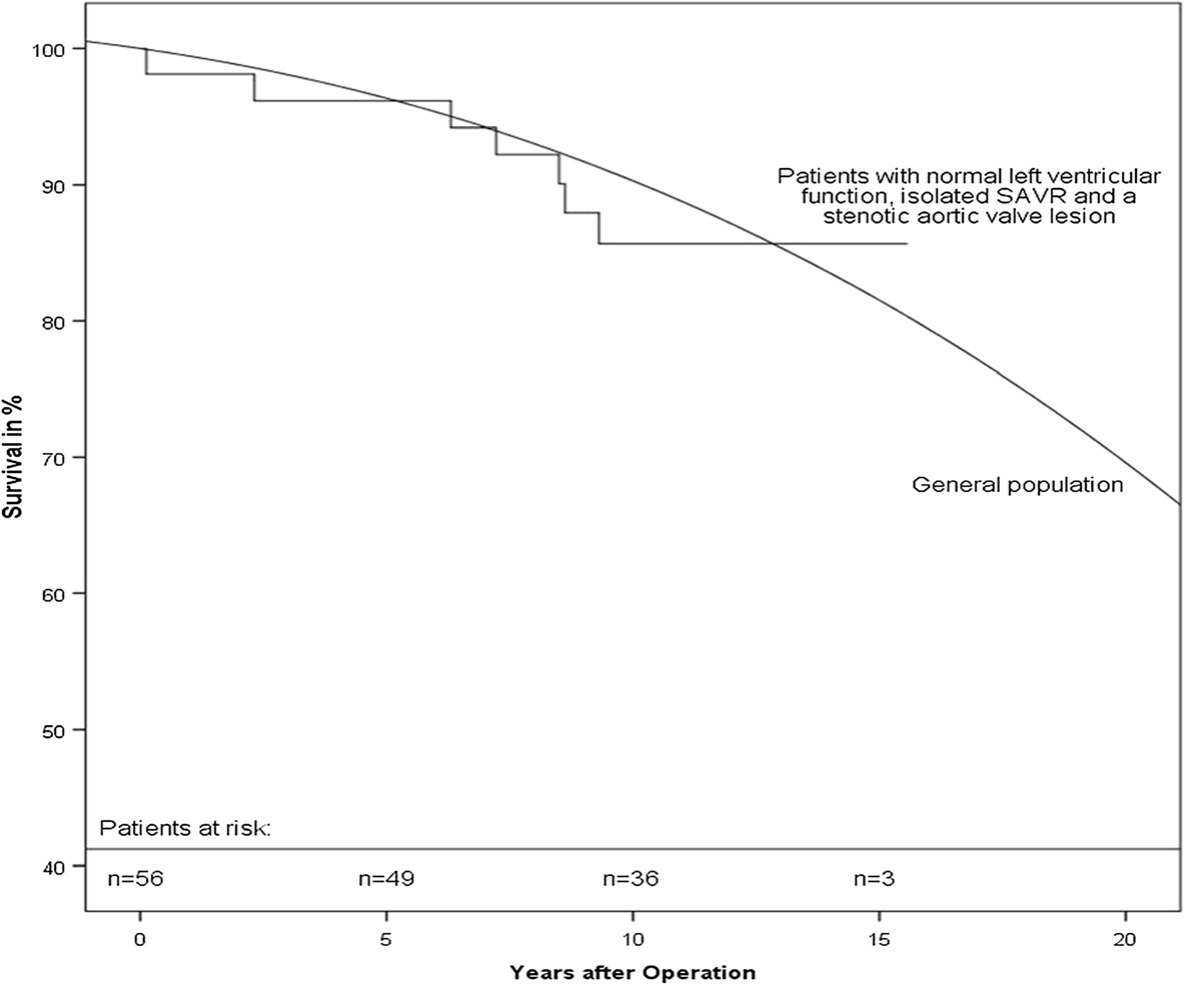
Survival after SAVR compared to general population.

The above mentioned problems in comparing different study cohorts also compromise the comparison to age- and gender-matched survival estimates from the German general population. To create a comparable cohort, only patients with isolated AVR and normal left ventricular function were analyzed, whereas patients with isolated aortic valve regurgitation as cause of operation were excluded because of the above mentioned impaired survival [9,10]. For the above described cohort a survival rate comparable to the general population was seen (Figure 3). One has to take into consideration that reported mortality rates of younger patients after AVR are higher than in the general population, while older patients reach the same survival rate as the general population [17].

Patients with impaired left ventricular function are especially demanding to compare. Survival in this cohort is substantially reduced. At 14.43 ± 0.54 years (median time of reoperation observed in this study), survival is at 40.6% ± 8.1%. After SAVR several authors reported a greater improvement of left ventricular function [8] and a resulting survival benefit [18], so that SAVR should be preferred in patients with impaired left ventricular function.

Because small SAVRs (≤25 mm) were associated with inferior survival in our study cohort, patient-prostheses mismatch (PPM) has to be discussed in this context. The concept of PPM was originally introduced by Rahimtoola [19] and describes an effective prosthetic valve area, which is smaller than that of a normal valve, usually defined as effective valve orifice area indexed per body surface (EOAI) smaller than 0.85 cm^2^/m^2^. PPM is not to be expected in larger valve sizes. But smaller valve sizes, especially stented bioprostheses smaller than 25 mm, can be associated with PPM. SAVRs usually fit much more into patients native aortic root compared with stented bioprostheses and offer larger orifice areas due to their stentless construction. Therefore, PPM should not occur in stentless bioprostheses and in a variety of studies EOAI identified no mismatch [20]. Similarly, no case of PPM could be identified in our cohort. Nonetheless, small sizes of SAVRs were related to lower survival. Potentially, there are other factors involved, e.g. the higher reoperation rate in patients with small SAVR could lower patients survival or there could be a direct impact of the lower valve orifice area on survival. But for such conclusion our study population (only 20.2% of the patients received SAVRs ≤25 mm) is not large enough and further studies are necessary.

Bioprosthetic AVR showed a time-dependent, decreased longevity, caused by structural valve deterioration [3]. This finally results in a rising need of reoperation. In 2006 the American Heart Association and the American College of Cardiology recommended the use of bioprosthetic aortic valve replacements in patients above the age of 65 years (depending on various other risk factors) [21]. An update in 2010 lowered the age to 60 years [4]. Banbury et al. showed that smaller sizes of bioprostheses have a tendency to lower durability [22]. Due to larger orifice area and less stress on the cusps of SAVR, their durability has been expected to be better than stented AVR. Study results comparing SAVR and stented AVR are controversial in terms of durability. Undoubtedly, the durability of the prostheses decreases along with decreasing age of patients [3]. Thus, the special dependence on the mean age of the study cohort complicates the comparison of studies. Besides this, most studies dealing with stented AVR only describe freedom from reoperation due to structural valve deterioration. In case of SAVR, with the importance of the surgical implantation itself for the function of the valve, overall freedom from reoperation is much more relevant in this study.

Welke et al. published a study on the Carpentier-Edwards pericardial AVR, where 58% freedom from explantation after 10 years was given for patients of 21 to 49 years of age, and of 68% for patients of 50 to 64 years of age [17]. For the same prostheses, age-stratified freedom from reoperation due to structural valve deterioration after 14 years in patients ≤65 years of age was around 35% [11]. After adding endocarditis and non- structural valve deterioration this rate may even be worse.

For the Hancock II AVR, Valfré et al. described a freedom from reoperation rate of 62.6% in a small cohort of patients ≤60 years (n = 50) at 15 years [12]. Rizzioli et al. published a 15 year freedom from reoperation rate of 55.8% for patients ≤60 years [23]. However, in both publications no mean age for the cohort is given.

Ruel et al. described a median time to reoperation of 10.2 years after implantation of stented AVR in a patient cohort with a mean age of 47.6 years [13]. Our patients had a mean age of 53.1 ± 7.1 years and the median time to reoperation was 14.43 ± 0.54 years.

Mechanical AVR showed lower reoperation rates. The Veterans Affairs Randomized Trial, for example, reports a reoperation-rate after mechanical AVR of 10% after 15 years [15]. However, this advantage is accompanied by the above mentioned problems [13].

The pulmonary autograft procedure was introduced by Ross in 1967 for young patients with aortic valve disease [24]. The operation is connected with concerns regarding late autograft competence and the consequences of creating pulmonary valve disease. However, long term results are promising [11,25]. Chambers et al. published data for the pulmonary autograft procedure. They reported a survival rate of the hospital survivors of 61%, freedom from autograft replacement of 75%, and freedom from replacement of pulmonary position homografts of 80% 20 years after operation [11]. However, one has to keep in mind that the mean age in this cohort was 32 years and the procedures were done between 1967 and 1984.

In recent years a rapid progression of percutaneous AVR can be seen. In Germany, 23.9% of isolated AVR was performed catheter-based in 2010 [26]. If long-term results are promising and this development persists we can assume that various reoperations can be performed catheter-based. This, however, is only possible after biological AVR.

### Limitations

The retrospective nature of this study design can lead to an underestimation of complication rates (endocarditis, thrombembolic events and bleeding etc.). Because it is likely that some events would not be captured, either because of patients´ misinterpretation of our questions or recall bias, some data were not surveyed. Also, NYHA functional class is subjective. Underestimation of symptom severity may lead to overestimation of potential clinical benefit in the statistical analysis. That is why these data were not analyzed. The different types of SAVR used in the researched cohort and pooling of the clinical results may have influenced the valve-related outcomes, even though the type of SAVR did not appear to be of statistical relevance.

Despite these limitations one has to consider the fact that a controlled randomized trial in young patients involving stentless, stented and mechanical AVR is ethically very difficult to perform, if not impossible.

## Conclusion

In respect of the mentioned limitations of the study and the comparison to other publications (to our knowledge no research for larger cohorts of patients ≤ 60 years with SAVR is published), our data show long-term results in survival which are comparable or even better than stented AVR and mechanical AVR. Freedom from reoperation is at least comparable to stented AVR. Mechanical AVRs show higher freedom from reoperation, but the risk of reoperation has to be weighed against the risk of lifelong anticoagulation treatment and stroke. In respect of the recent and rapid development in the field of catheter-based AVR, one should also be aware that after mechanical AVR, patients are deprived of this therapeutic option. Comparison of SAVR to the pulmonary autograft procedure in terms of survival is limited due to different mean age of study populations. In terms of freedom from reoperation, the pulmonary autograft procedure is superior.

We conclude that SAVR is a good and safe alternative to mechanical prostheses in patients aged ≤ 60 years and should be considered preferably in patients with impaired left ventricular function.

## Abbreviations

AVR: Aortic valve replacement; SAVR: Stentless aortic valve replacement; PPM: Patient prosthesis mismatch; EOAI: Effective valve orifice area indexed per body surface.

## Competing interests

The authors report no competing interests.

## Authors’ contributions

TC and HG designed the study; TC performed the statistical analysis and drafted the manuscript; BC was involved in collecting data and drafting the manuscript; WK and HG helped to draft the manuscript; WK, HG and BC gave critical comments on the results. All authors read and approved the final manuscript.
